# Comparing Dynamic Causal Models using AIC, BIC and Free Energy

**DOI:** 10.1016/j.neuroimage.2011.07.039

**Published:** 2012-01-02

**Authors:** W.D. Penny

**Affiliations:** Wellcome Trust Centre for Neuroimaging, University College, London WC1N 3BG, UK

**Keywords:** Bayesian, Model comparison, Brain connectivity, Dynamic Causal Modelling, fMRI

## Abstract

In neuroimaging it is now becoming standard practise to fit multiple models to data and compare them using a model selection criterion. This is especially prevalent in the analysis of brain connectivity. This paper describes a simulation study which compares the relative merits of three model selection criteria (i) Akaike's Information Criterion (AIC), (ii) the Bayesian Information Criterion (BIC) and (iii) the variational Free Energy. Differences in performance are examined in the context of General Linear Models (GLMs) and Dynamic Causal Models (DCMs). We find that the Free Energy has the best model selection ability and recommend it be used for comparison of DCMs.

## Highlights

► The Free Energy is a better model comparison criterion than AIC or BIC ► The complexity of a model is not usefully characterised by the number of parameters. ► Empirical Bayesian estimation of prior variances would improve model comparison.

## Introduction

Mathematical models of scientific data can be formally compared using the Bayesian model evidence (Bernardo and Smith, 2000; Gelman et al., 1995; Mackay, 2003), an approach that is now widely used in statistics (Hoeting et al., 1999), signal processing (Penny and Roberts, 2002), machine learning (Beal and Ghahramani, 2003), and neuroimaging (Friston et al., 2008; Penny et al., 2003; Trujillo-Barreto et al., 2004). By comparing the evidence or ‘score’ of one model relative to another, a decision can be made as to which is the more veridical. This approach has now been widely adopted for the analysis of brain connectivity data, especially in the context of Dynamic Causal Modelling (DCM) (Friston et al., 2003; Penny et al., 2004).

Originally (Penny et al., 2004), it was proposed to score DCMs using a combination of Akaike's Information Criterion (AIC) and the Bayesian Information Criterion (BIC) criteria. Specifically, it was proposed that (Penny et al., 2004) if both AIC and BIC provided a log Bayes factor (difference in log model evidences) of greater than three in favour of model one versus two, one could safely conclude that model one was the more veridical. More recently it has been proposed (Stephan et al., 2010), on theoretical grounds, to instead score DCMs using the Free Energy (Friston et al., 2007a). However, until now there has been no empirical comparison of the model comparison abilities of the different approaches.

This motivates the work in this paper which describes a simulation study comparing AIC, BIC and the Free Energy. Differences in performance are examined in the context of General Linear Models (GLMs) and Dynamic Causal Models (DCMs). Specifically, for each class of model we define a ‘full’ and a ‘nested’ model, where the nested model is a special case of the full model with a subset of parameters set to zero. We examine how model comparison accuracy varies as a function of number of data points and signal to noise ratio for the separate cases of data being generated by full or nested models. This allows us to assess the sensitivity and specificity of the different model comparison criteria. The paper uses simulated data generated from models with known parameters but these parameters are derived from empirical neuroimaging data. We start by briefly reviewing the relevant theoretical background and then go on to present our results.

## Methods

We consider Bayesian inference on data *y* using model *m* with parameters *θ*. In the analysis of brain connectivity, the data would comprise, for example, fMRI time series from multiple brain regions, the model would make specific assumptions about connectivity structure, and the parameters would correspond to connections strengths. A generic approach for statistical inference in this context is Bayesian estimation (Bishop, 2006; Gelman et al., 1995) which provides estimates of two quantities. The first is the posterior distribution over model parameters *p*(*θ*|*m*, *y*) which can be used to make inferences about model parameters *θ*. The second is the probability of the data given the model, otherwise known as the model evidence. This can be used for model comparison, in that ratios of model evidences (Bayes factors) allow one to choose between models (Kass and Raftery, 1995; Raftery, 1995). This paper focusses on Dynamic Causal Models and on model inference using AIC, BIC or Free Energy approximations to the model evidence. We first describe DCM, show how model parameters are estimated, describe Bayesian inference for General Linear Models and then go on to describe the different model selection criteria. In what follows N (*x* ; *m*, *S*) represents a multivariate Gaussian density over *x* with mean *m* and covariance *S*, and |*S*| denotes the determinant of matrix *S*.

### DCM for fMRI

Dynamic Causal Modelling is a framework for fitting differential equation models of neuronal activity to brain imaging data using Bayesian inference. There is now a library of DCMs and variants differ according to their level of biological realism and the data features which they explain. The DCM approach can be applied to functional Magnetic Resonance Imaging (fMRI), Electroencephalographic (EEG), Magnetoencephalographic (MEG), and Local Field Potential (LFP) data (Daunizeau et al., 2009). The empirical work in this paper uses DCM for fMRI.

#### Neurodynamics

This paper uses DCM for fMRI with bilinear neurodynamics and an extended Balloon model (Friston, 2002) for the hemodynamics. The neurodynamics are described by the following multivariate differential equation

(1) $${\dot{z}}_{t}=\left(A+\sum_{j=1}^{M}\;u_{t}(j)B^{j}\right)z_{t}+Cu_{t}$$

where *t* indexes continuous time and the dot notation denotes a time derivative. The *i*th entry in *z*_*t*_ corresponds to neuronal activity in the *i*th brain region, and *u*_*t*_(*j*) is the *j*th experimental input.

A DCM is characterised by a set of ‘intrinsic connections’, *A*, that specify which regions are connected and whether these connections are unidirectional or bidirectional. We also define a set of input connections, *C*, that specify which inputs are connected to which regions, and a set of modulatory connections, *B*^*j*^, that specify which intrinsic connections can be changed by which inputs. Usually, the *B* parameters are of greatest interest as these describe how connections between brain regions are dependent on experimental manipulations.

The overall specification of input, intrinsic and modulatory connectivity comprise our assumptions about model structure. This in turn represents a scientific hypothesis about the structure of the large-scale neuronal network mediating the underlying cognitive function. These hypotheses, or models are indexed by *m*.

The simulations in this paper use ‘DCM 8’ (available in SPM8 prior to revision 4010) with a deterministic, single-state, bilinear neurodynamical model as described above.

#### Model predictions

In DCM, neuronal activity gives rise to fMRI signals via an extended Balloon model (Buxton et al., 2004) and BOLD signal model (Stephan et al., 2007) for each region. This specifies how changes in neuronal activity give rise to changes in blood oxygenation that are measured with fMRI. The equations for these hemodynamics are provided in the Appendix A and depend on a set of hemodynamic parameters *h*.

Overall, the DCM parameters are collectively written as the vector *θ* = {*A*, *B*, *C*, *h*}. Numerical integration of the neurodynamic (Eq. 1) and hemodynamic equations (Appendix A) leads to prediction of fMRI activity in each brain region. These values are concatenated to produce a single model prediction vector *g*(*θ*).

#### Priors

The priors factorise over parameter types

(2) p(θ|m)=p(A|m)p(B|m)p(C|m)p(h|m)

and each parameter prior is Gaussian. The priors used in this paper correspond to those used in ‘DCM8’. The priors over the intrinsic connections are chosen to encourage stable dynamics. This results in prior variances which depend on the number of regions in the model (Friston et al., 2003), and in this paper we model activity in three regions. For the intrinsic self-connections we have

(3) $$p(A_{\mathrm{ii}}|m)=\text{N }(A_{\mathrm{ii}};-1,\sigma_{\mathrm{self}}^{2})$$

with *σ*_*self*_ = 0.177. The time constant associated with a self-connection is *τ*_*i*_ = − 1/*A*_*ii*_, and the time at which activity decays to half its initial value (half-life) is (1/*A*_*ii*_)*log*0.5 (Friston et al., 2003). The prior over self-connections corresponds to a prior over half-life's that can be determined by sampling from *p*(*A*_*ii*_|*m*) and transforming variables to *τ*_*i*_ = − 1/*A*_*ii*_. This produces a mean half life of approximately 720 ms with 90% of the distribution between 500 and 1000 ms.

For those intrinsic cross connections which are not fixed at zero by model assumptions *m* we have

(4) $$p(A_{\mathrm{ik}}|m)=\text{N }(A_{\mathrm{ik}};1/64,\sigma_{\mathrm{cross}}^{2})$$

where *σ*_*cross*_ = 0.5. Elements of the modulatory and input connectivity matrices (which are not fixed at zero by model assumptions) have shrinkage priors

(5) p(Bikj|m)=N(Bikj;0,σs2)

(6) p(Cij|m)=N(Cij;0,σs2)

and *σ*_*s*_ = 2. In the above, *i* and *k* index brain regions and *j* indexes experimental input.

The prior variance parameters *σ*_*self*_^2^, *σ*_*cross*_^2^ and *σ*_*s*_^2^ along with the prior variances on hemodynamic parameters (see Appendix A) determine the overall prior covariance on model parameters, *C*_*θ*_ (see next section). In the free energy model comparison criterion (see below) these variances contribute to the penalty paid for each parameter.

### Optimisation

The standard algorithm used to optimise DCMs is the Variational Laplace (VL) method described in (Friston et al., 2007a). The VL algorithm can be used for Bayesian estimation of any nonlinear model of the form

(7) y=g(θ)+e

where *g*(*θ*) is some nonlinear function, and *e* is zero mean additive Gaussian noise with covariance *C*_*y*_. This covariance depends on hyperparameters *λ* as shown below. The likelihood of the data is therefore

(8) $$p(y|\theta ,\lambda ,m)=\text{N}(y;g(\theta ,m),C_{y})$$

The framework allows for Gaussian priors over model parameters

(9) $$p(\theta |m)=\text{N}(\theta ;\mu_{\theta },C_{\theta })$$

where the prior mean and covariance are assumed known. The error covariances are assumed to decompose into terms of the form

(10) $$C_{y}^{-1}=\sum_{i}\;exp(\lambda_{i})Q_{i}$$

where *Q*_*i*_ are known precision basis functions. The hyperparameters that govern these error precisions are collectively written as the vector *λ*. These will be estimated. Additionally, the hyperparameters are constrained by the prior

(11) $$p(\lambda |m)=\text{N}(\lambda ;\mu_{\lambda },C_{\lambda })$$

The above distributions allow one to write down an expression for the joint log likelihood of the data, parameters and hyperparameters

(12) p(y,θ,λ|m)=p(y|θ,λ,m)p(θ|m)p(λ|m)

The VL algorithm then assumes an approximate posterior density of the following factorised form

(13) $$\begin{array}{c} q(\theta ,\lambda |y,m)=q(\theta |y,m)q(\lambda |y,m)\\ q(\theta |y,m)=\text{N}(\theta ;m_{\theta },S_{\theta })\\ q(\lambda |y,m)=\text{N}(\lambda ;m_{\lambda },S_{\lambda }) \end{array}$$

The parameters of these approximate posteriors are then iteratively updated so as to minimise the Kullback–Liebler (KL)-divergence between the true and approximate posteriors. This algorithm is described fully in (Friston et al., 2007a).

We emphasise here that the Variational Laplace framework assumes that the prior means and covariances (*μ*_*θ*_, *C*_*θ*_, *μ*_*λ*_, *C*_*λ*_) are known. They are not estimated from data, as is the case for Empirical Bayes methods (Carlin and Louis, 2000). We will return to this issue in the discussion.

#### Hyperparameters in DCM for fMRI

In DCM for fMRI the precision basis functions *Q*_*i*_, defined in Eq. (10), are set to *Q*_*i*_ = *I* for each brain region. The quantity *γ*_*i*_ = *exp*(*λ*_*i*_) therefore corresponds to the noise precision in region *i*.

The overall error covariance matrix *C*_*y*_ has a block structure corresponding to the assumption that observation noise is independent and identically distributed in each region. This is valid as time series data are usually pre-whitened before entering into a DCM analysis (Friston et al., 2003). The prior mean and covariance of the associated latent variables are set to

(14) $$\begin{array}{c} \mu_{\lambda }=0\\ C_{\lambda }=1 \end{array}$$

This corresponds to the assumption that the mean prior noise precision, ${\bar{\gamma }}_{i}=1.7$. These values, along with the priors on the neurodynamic parameters, have been set so as to produce data sets with typical signal to noise ratios encountered in fMRI.

### Model evidence

The model evidence, also known as the marginal likelihood, is not straightforward to compute, since its computation involves integrating out the dependence on model parameters

(15) p(y|m)=∫∫p(y|θ,λ,m)p(θ|m)p(λ|m)dθdλ.

The following sections describe Free Energy, AIC and BIC approximations to the (log) model evidence. Once the evidence has been computed models *m*_1_ and *m*_2_ can be compared using the Bayes factor

(16) $$B_{12}=\frac{p(y|m_{1})}{p(y|m_{2})}$$

with a value of 20 corresponding to a posterior probability of greater than 0.95 in favour of model *m*_1_. The corresponding log Bayes factor is 3. The use of Bayes factors for model comparison is described more fully elsewhere (Kass and Raftery, 1995; Penny et al., 2004). Comparison of a large number of models is best implemented using the full posterior density, *p*(*m*|*y*), as described in (Penny et al., 2010).

### Free energy

It is possible to place a lower bound on the log model evidence of the following form (Beal, 2003)

(17) logp(y|m)=F(m)+KL[q(θ,λ|m)||p(θ,λ|y,m)]

where *F(m)* is known as the negative variational free energy (henceforth ‘Free Energy’) and the last term is the Kullback–Liebler distance between the true posterior density, *p*(*θ*, *λ*|*y*, *m*) and an approximate posterior *q*(*θ*, *λ*|*m*). Because *KL* is always positive (Mackay, 2003), *F(m)* provides a lower bound on the model evidence.

The Free Energy is defined as

(18) $$F(m)=\int \int q(\theta ,\lambda |y,m)log\left[\frac{p(y,\theta ,\lambda |m)}{q(\theta ,\lambda |y,m)}\right]\;d\theta d\lambda$$

and can be estimated using a Laplace approximation (Friston et al., 2007a), *F*_*L*_(*m*), as derived in Appendix B. As noted in (Wipf and Nagarajan, 2009), because the Laplace approximation is not exactly equal to the Free Energy, the above lower bound property no longer holds. That is, the Laplace approximation does not lower bound the log model evidence. As we shall see, however, it nevertheless provides a very useful model comparison criterion. The Laplace approximation to the Free Energy is given in Eq. (57) and can be expressed as a sum of accuracy and complexity terms (Beal, 2003)

(19) $$F_{L}(m)=Accuracy(m)-Complexity(m)$$

(20) $$Accuracy(m)=-\frac{1}{2}e_{y}^{T}C_{y}^{-1}e_{y}-\frac{1}{2}log|C_{y}|-\frac{N}{2}log2\pi$$

(21) $$\begin{array}{l} Complexity(m)=\frac{1}{2}e_{\theta }^{T}C_{\theta }^{-1}e_{\theta }+\frac{1}{2}log|C_{\theta }|-\frac{1}{2}log|S_{\theta }|\\ +\frac{1}{2}e_{\lambda }^{T}C_{\lambda }^{-1}e_{\lambda }+\frac{1}{2}log|C_{\lambda }|-\frac{1}{2}log|S_{\lambda }| \end{array}$$

where *N* is the number of data points and the ‘error terms’ are

(22) $$\begin{array}{l} e_{y}=y-g(m_{\theta })\\ e_{\theta }=m_{\theta }-\mu_{\theta }\\ e_{\lambda }=m_{\lambda }-\mu_{\lambda } \end{array}$$

The first row of Eq. (21) is the complexity term for the parameters and the second row the complexity term for the hyperparameters. If the hyperparameters are known then the last row of Eq. (21) disappears. In this case we can write the complexity as

(23) $$Complexity(m)=\frac{1}{2}e_{\theta }^{T}C_{\theta }^{-1}e_{\theta }+\frac{1}{2}log\frac{\left|C_{\theta }\right|}{\left|S_{\theta }\right|}$$

In the limit that the posterior equals the prior (*e*_*θ*_ = 0,*C*_*θ*_ = *S*_*θ*_), the complexity term equals zero. The last term in Eq. (23), $\frac{1}{2}log\frac{\left|C_{\theta }\right|}{\left|S_{\theta }\right|}$, is also referred to as an Occam factor (see page 349 in (Mackay, 2003)). Because the determinant of a matrix corresponds to the volume spanned by its eigenvectors, this Occam factor gets larger and the model evidence smaller as the posterior volume, |*S*_*θ*_|, reduces in proportion to the prior volume, |*C*_*θ*_|. Models for which parameters have to be specified precisely (small posterior volume) are brittle. They are not good models (complexity is high).

The above considerations also apply to cases where hyperparameters are estimated. There is an additional complexity term (last line of Eq. 21) and therefore an additional Occam factor.

#### Correlated parameters

Other factors being equal, models with strong correlation in the posterior are not good models. For example, given a model with just two parameters the determinant of the posterior covariance is given by

(24) $$|S_{\theta }|=(1-r^{2})\sigma_{\theta_{1}}^{2}\sigma_{\theta_{2}}^{2}$$

where *r* is the posterior correlation, *σ*_*θ*_1__ and *σ*_*θ*_2__ are the posterior standard deviations of the two parameters. For the case of two parameters having a similar effect on model predictions the posterior correlation will be high, therefore implying a large complexity penalty.

However, there is another factor at play. This is that neither parameter will be estimated accurately (the posterior variances will be high). This second factor can offset the higher complexity due to correlation and can lead to a situation in which additional extraneous parameters will not lead to a significant drop in free energy. One would then appeal to a further Occam's Razor principle (Mackay, 2003), namely, that in the absence of significant free energy differences one should prefer the simpler model (see Discussion).

When fitting DCMs to fMRI data it is likely that some parameters will be correlated with each other. This correlation can be examined by looking at the posterior covariance matrix *S*_*θ*_. A good example of this is provided in Fig. 6 of Stephan et al. (2007) who describe posterior correlations among hemodynamic and connectivity parameters. Importantly, these correlations are accomodated in the Free Energy model comparison criterion (see Eq. 23 and above). This is possible because Variational Laplace does not assume that parameters are a posteriori independent among themselves, rather it is assumed that the parameters are a posteriori independent of the hyperparameters (see Eq. 13).

#### Decompositions

It is instructive to decompose approximations to the model evidence into contributions from specific sets of parameters or predictions. In the context of DCM, one can decompose the accuracy terms into contributions from different brain regions, as described previously (Penny et al., 2004). This enables insight to be gained into why one model is better than another. It may be, for example, that one model predicts activity more accurately in a particular brain region.

Similarly, it is possible to decompose the complexity term into contributions from different sets of parameters. If we ignore correlation among different parameter sets then the complexity is approximately

(25) $$Complexity(m)\approx \frac{1}{2}\sum_{j}\;\left(e_{\theta_{j}}^{T}C_{\theta_{j}}^{-1}e_{\theta_{j}}+log\frac{\left|C_{\theta_{j}}\right|}{\left|S_{\theta_{j}}\right|}\right)$$

where *j* indexes the *j*th parameter set. In the context of DCM these could index input connections (*j* = 1), intrinsic connections (*j* = 2), modulatory connections (*j* = 3) etc. We will see an example of this in the Results section.

#### General Linear Models

For General Linear Models (GLMs) model predictions are given by

(26) g(θ)=Xθ

where *X* is a design matrix and *θ* are now regression coefficients. The posterior distribution is analytic and given by (Bishop, 2006)

(27) $$\begin{array}{c} S_{\theta }^{-1}=X^{T}C_{y}^{-1}X+C_{\theta }^{-1}\\ m_{\theta }=S_{\theta }\left(X^{T}C_{y}^{-1}y+C_{\theta }^{-1}\mu_{\theta }\right) \end{array}$$

These parameter values can then be plugged into Eqs. (19) to (22) to compute the Free Energy. If the hyperparameters are assumed known then the Free Energy expression in Eq. (19) is exactly equal to the log model evidence.That is, *F*_*L*_(*m*) = *logp*(*y*|*m*). We will revisit this case in the Results section. If the hyperparameters are estimated then the Free Energy provides a very close approximation, as confirmed by sampling methods (Friston et al., 2007a).

### AIC and BIC

A simple approximation to the log model evidence is given by the Bayesian Information Criterion (Schwarz, 1978)

(28) $$BIC=Accuracy(m)-\frac{p}{2}logN$$

where *p* is the number of parameters, and *N* is the number of data points. The BIC is a special case of the Free Energy approximation that drops all terms that do not scale with the number of data points (see e.g. Appendix A2 in (Penny et al., 2004) for a derivation). This is equivalent to the statement that BIC is equal to the Free Energy under the infinite data limit, and when the priors over parameters are flat, and the variational posterior is exact (see section 2.3 in (Attias, 1999) and page 217 in (Bishop, 2006)). In practise, as we shall see, these three requirements are almost never met and BIC will produce model comparisons that are often very different to those from the Free Energy.

An alternative model selection criterion is Akaike's Information Criterion (or ‘An Information Criterion’) (Akaike, 1973)

(29) AIC=Accuracy(m)−p

AIC is not a formal approximation to the model evidence but derives from information theoretic considerations. Specifically, AIC model selection will choose that model in the comparison set with minimal expected KL divergence to the true model (Akaike, 1973; Burnham and Anderson, 2002). There are precedents in the literature, however, for using it as a surrogate for the model evidence, in order to derive a posterior density over models (Burnham and Anderson, 2004) (Penny et al., 2004).

The AIC criterion has been reported to perform poorly for small numbers of data points (Brockwell and Davis, 2009; Burnham and Anderson, 2004). This has motivated the inclusion of a correction term

(30) $$AICc=AIC-\frac{p(p+1)}{N-p-1}$$

known as the ‘corrected’ AIC (AICc) (Hurvich and Tsai, 1989). The AICc criterion thus penalises parameters more than does AIC. The two criteria become approximately equal for *N* > *p*^2^ and identical in the limit of very large sample sizes. We note, however, that for *N* < *p* + 1 the denominator in the correction term becomes negative and AICc penalises parameters less than does AIC. In the empirical work in this paper we therefore avoid this (highly unlikely) regime.

In applications of AIC and BIC to DCMs (Penny et al., 2004), the estimated parameters are taken to be equal to the posterior means *m*_*θ*_ and *m*_*λ*_. AIC and BIC are useful approximations because one only needs to quantify the fit of the model to provide an estimate of the log-evidence. AIC and BIC are qualitatively different to the free energy approximation in that the same fixed penalty is paid for each parameter in the model.

## Results

### Linear models

We first compare the different approximations to the model evidence using Bayesian GLMs. We define these using the following prior and likelihood

(31) $$\begin{array}{l} \;p(\theta )=N(\theta ;\mu_{\theta },C_{\theta })\\ p(y|\theta )=N(y;X\theta ,C_{y}) \end{array}$$

where *θ* is the [*p* × 1] vector of regression coefficients, *y* is the [*N* × 1] vector of data points, *X* is the [*N* × *p*] design matrix, and for the prior mean we have *μ*_*θ*_ = 0. For the work in this paper we assume isotropic covariance matrices

(32) $$\begin{array}{l} C_{\theta }=\sigma_{p}^{2}I_{p}\\ C_{y}=\sigma_{e}^{2}I_{N} \end{array}$$

where *σ*_*p*_ and *σ*_*e*_ are the standard deviations of the prior and observation error. We assume that these parameters are known.

We compare Bayes factors based on AIC, BIC and *F*_*L*_ for nested GLMs derived from an fMRI study. The fMRI data set was collected to study neuronal responses to images of faces and is available from the SPM web site (http://www.fil.ion.ucl.ac.uk/spm/data/face_rep/face_rep_SPM5.html.). Each face was presented twice, and faces either belonged to familiar or unfamiliar people. This gave rise to four conditions, each of which was modelled with 3 hemodynamic basis functions (Friston et al., 2007b). For a full description of this data set and similar analyses see (Henson et al., 2002).

**Fig. 1:**
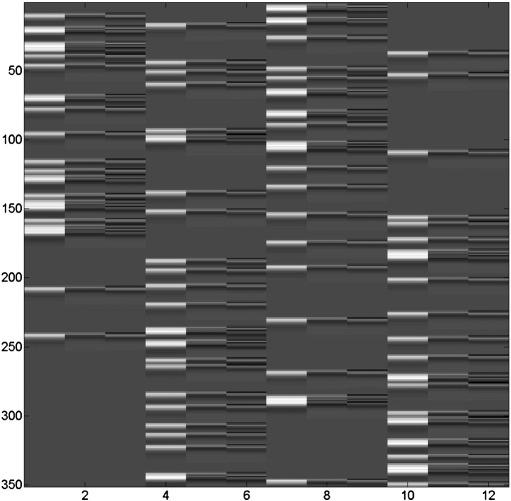
Design matrix for the full GLM. The nested GLM uses an identical design matrix but with the first three columns removed. The full design matrix comprises *N* = 351 rows, one for each fMRI scan, and twelve columns, one for each putative experimental effect.

We first define a ‘nested’ model in which only 3 of these conditions are modelled, resulting in 9 regressors. We then define a ‘full’ model as containing an extra 3 regressors from the additional condition (first response to unfamiliar faces). Fig. 1 shows the design matrix for the full model. The design matrices for the full and nested models are therefore different, with the full model design matrix having 12 regressors and the nested model having 9 regressors.

Estimated regression coefficients, $\hat{\theta }$, and noise variance estimates, *σ̂*_*e*_ = 0.73 were extracted for a voxel showing a significant overall response to faces (i.e. over all conditions). The corresponding fMRI time series comprised *N* = 351 values. We then created simulated data based on this observed fMRI data as follows.

First, we estimated the deviation of the fitted regression coefficients about zero and set the prior SD to this value, *σ*_*p*_ = 6.05. This estimation was based on parameter fits from data at a single voxel. The use of a common *σ*_*p*_ value for all regression coefficients implies that the effects are of similar magnitude for all four conditions and all three temporal basis functions, and is a reasonable assumption. We then computed < *σ*_*y*_ >, the average signal standard deviation when drawing parameters the prior *p*(*θ*).

We then produced simulated data sets where the Signal to Noise ratio

(33) $$SNR=\frac{<\sigma_{y}>}{\sigma_{e}}$$

was set to a range of values by choosing an appropriate *σ*_*e*_. SNR defined in this manner can be related to the proportion of variance explained by the model, as shown in Appendix C. The observed fMRI data have a value of *SNR* = 1.3.

Each simulated data set was then generated by drawing regression coefficients from their prior densities, producing model predictions *g* = *Xθ* (for both full and nested models) and adding zero mean Gaussian noise with variance *σ*_*e*_^2^.

We then fitted both full and nested models to each simulated data set and estimated Bayes factors using AIC, BIC and *F*_*L*_. These criteria were computed by substituting *X*, *C*_*y*_, *C*_*θ*_, and *μ*_*θ*_ as defined in this section into Eq. (27) for computing the posterior mean and covariance for linear models. The prediction errors, *e*_*y*_, and parameter errors, *e*_*θ*_, were then computed from Eqs. (22) and (26). We could then compute the accuracy and complexity terms using Eqs. (20) and (21) (the complexity terms for *λ* were ignored as the observation noise variance was known for these simulations).

**Fig. 2:**
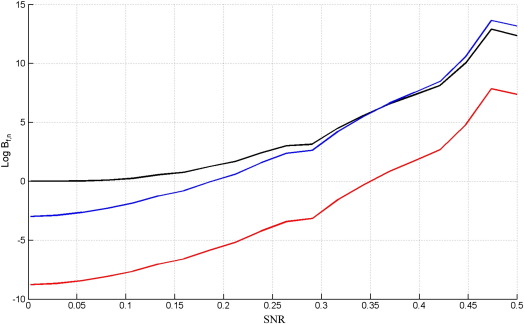
Log Bayes factor of full versus nested model, Log *B*_*f*, *n*_, versus the signal to noise ratio, SNR, when the true model is the full GLM for *F*_*L*_ (black), AIC (blue) and BIC (red).

Fig. 2 shows results for data drawn from the full model. The figure plots the log Bayes factors (differences in log model evidence) at various values of *SNR*, where each point in each curve was averaged over 1000 simulated data sets. At low SNRs, experimental effects should be impossible to detect. This is reflected in the Free Energy log Bayes factor which correctly asymptotes to a value of zero, indicating neither model is preferred. In this regime, however, BIC and to a lesser extent AIC both incorrectly favour the nested model. The error bars on the plots (not shown) are extremely tight in this regime, being ± 0.0001, ± 0.09 and ± 0.35 for SNRs of 0.0025, 0.029 and 0.055 respectively (averaged over the three criteria). This means we can be highly confident that *F*_*L*_ is unbiased but that AIC and BIC are biassed towards the nested model.

**Fig. 3:**
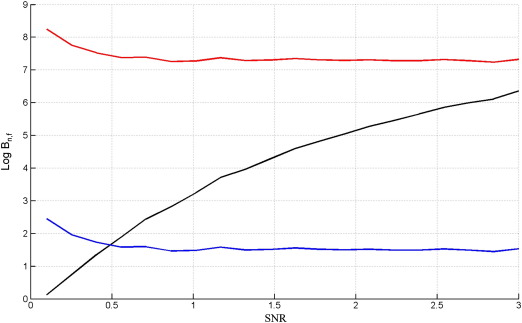
Log Bayes factor of nested versus full model, Log *B*_*n*, *f*_, versus the signal to noise ratio, SNR, when the true model is the nested GLM for *F*_*L*_ (black), AIC (blue) and BIC (red).

The above procedure was then repeated but this time generating data from the nested model. The results are shown in Fig. 3 (note the broader range of SNRs plotted). In the low SNR regime, model comparison should again be impossible. This is correctly reflected in the *F*_*L*_ criterion with a log Bayes factor approaching zero, but not so in the AIC or BIC criteria.

**Fig. 4:**
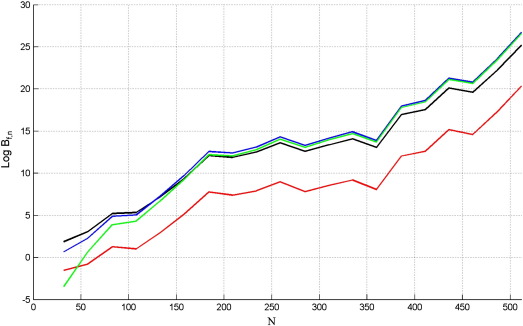
Log Bayes factor of full versus nested model, Log *B*_*f*, *n*_, versus the number of data points, *N*, when the true model is the full GLM for *F*_*L*_ (black), AIC (blue), BIC (red) and AICc (green).

Finally, we examined the dependence of model comparison on the number of data points, *N*. We varied *N* over 20 values between 32 and 512 with 1000 replications at each value, using *SNR* = 0.5 (results were qualitatively similar for other SNRs). The results are shown in Fig. 4 for data generated from the full model. As expected, Bayes factors increase with the number of data points. The free energy, AIC and AICc show very similar performance with *F*_*L*_ being slightly better at low *N* and AIC/AICc at high *N*. The BIC criterion is biassed towards the nested model.

**Fig. 5:**
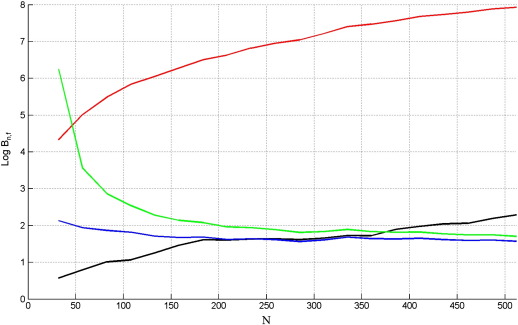
Log Bayes factor of nested versus full model, Log *B*_*n*, *f*_, versus the number of data points, *N*, when the true model is the nested GLM for *F*_*L*_ (black), AIC (blue), BIC (red) and AICc (green).

Fig. 5 shows the results for data generated from the nested model. The Bayes factors from the free energy and BIC increase with the number of data points, whereas this is not the case for AIC and AICc. We see that AIC and AICc are equivalent for large sample sizes. For small sample sizes AICc pays a larger parameter penalty. This is beneficial when the nested model is true (Fig. 5) but not when the full model is true (Fig. 4). Overall, we do not see a good reason for favouring AICc over AIC and so exclude it from subsequent model comparisons.

Theory (Attias, 1999) tells us that BIC should converge to the Free Energy for large sample sizes. However, this is only the case for flat priors over parameters and if the variational posterior is correct. As we have linear models, the last requirement is met but the prior over parameters is Gaussian, rather than flat. A data set comprising 512 points is about the maximum one could hope to get from a single session of fMRI scanning (approximately 17 min with a TR of 2s). We therefore conclude that for neuroimaging applications BIC and Free Energy are likely to give different results.

### DCM for fMRI

We now compare the model comparison criteria using DCM for fMRI. We generate data using synthetic DCMs with known parameter values. However, to ensure the data are realistic we use parameter values that were estimated from neuroimaging data.

This data derive from a previously published study on the cortical dynamics of intelligible speech (Leff et al., 2008). We used data from a single representative subject. This study applied DCM for fMRI to investigate activity among three key multimodal brain regions: the left posterior and anterior superior temporal sulci (subsequently referred to as regions P and A respectively) and pars orbitalis of the inferior frontal gyrus (region F). The aim of the study was to see how connections among regions depended on whether the auditory input was intelligible speech or time-reversed speech. Full details of the experimental paradigm and imaging parameters are available in (Leff et al., 2008). The time series which were modelled in this study comprise *N* = 488 data points in each of three brain regions.

**Fig. 6:**
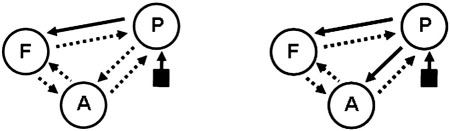
A nested (left) and full (right) DCM. The full DCM is identical to the nested DCM except for having an additional modulatory forward connection from region P to region A. Intrinsic connections are indicated by dotted arrows, modulatory connections by overlaid solid arrows and inputs by solid squares with an arrow.

We focus on just two of the models considered by Leff et al. (Leff et al., 2008). These are a ‘nested’ model, which has full intrinsic connectivity with auditory input, *u*_*aud*_, entering region P, and a modulatory connection from region P to F (this allows region F to be differentially responsive to intelligible versus time-reversed speech). We also define a ‘full’ model which is identical but has an additional modulatory connection from region P to A (*b*_*AP*_ — see below). The two networks are shown in Fig. 6. The two models differ in only a single connection and we chose these very similar models to make model comparison as challenging as possible.

Mathematically, neurodynamics evolve according to

(34) $$\begin{array}{c} \left[\begin{array}{c} {\dot{z}}_{P}\\ {\dot{z}}_{F}\\ {\dot{z}}_{A} \end{array}\right]=\left(\left[\begin{array}{ccc} a_{\mathrm{PP}} & a_{\mathrm{PF}} & a_{\mathrm{PA}}\\ a_{\mathrm{FP}} & a_{\mathrm{FF}} & a_{\mathrm{FA}}\\ a_{\mathrm{AP}} & a_{\mathrm{AF}} & a_{\mathrm{AA}} \end{array}\right]+u_{\mathrm{int}}\left[\begin{array}{ccc} 0 & 0 & 0\\ b_{\mathrm{FP}} & 0 & 0\\ b_{\mathrm{AP}} & 0 & 0 \end{array}\right]\right)\left[\begin{array}{c} z_{P}\\ z_{F}\\ z_{A} \end{array}\right]\\ +u_{\mathrm{aud}}\left[\begin{array}{c} c_{P}\\ 0\\ 0 \end{array}\right] \end{array}$$

where *u*_*aud*_ is a train of auditory input spikes, *u*_*int*_ indicates whether the input is intelligible (Leff et al., 2008), *a*_*AF*_ denotes the value of the intrinsic connection from region *F* to *A*, *b*_*FP*_ and *b*_*AP*_ are the strengths of the two modulatory connections, and *c*_*P*_ is the strength of the input connection. For the nested DCM we have *b*_*AP*_ = 0.

We first generated data sets from the full model over a range of SNRs as follows. To best reflect the empirical fMRI data all parameters other than the modulatory parameters were held constant. For each simulated data set the modulatory parameters were first drawn from their prior densities (see Eq. 5). Additionally, the modulatory parameters were then constrained to be positive (by taking the absolute value) so that modulatory effects would be facilitating.

Synthetic fMRI data was then generated by integrating the neurodynamic and hemodynamic equations and adding observation noise to obtain the target SNR. The SNR was defined in the same way as for the linear models, but with the signal standard deviation, < *σ*_*y*_ >, averaged over the three predicted time series (one for each brain region). The observed fMRI data have a value of SNR = 0.2. We then fitted both full and nested models to each simulated data set and estimated Bayes factors using AIC, BIC and *F*_*L*_.

**Fig. 7:**
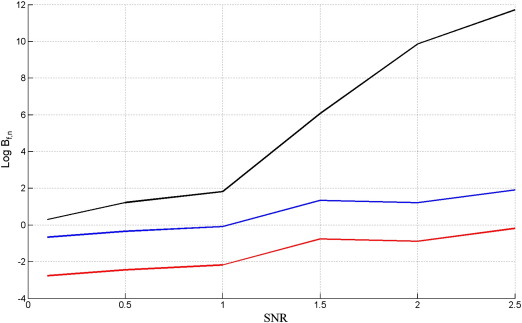
Log Bayes factor of full versus nested model, Log *B*_*f*, *n*_, versus the signal to noise ratio, SNR, when the true model is the full DCM for *F*_*L*_ (black), AIC (blue) and BIC (red).

Fig. 7 shows results for data drawn from the full model. The figure plots the log Bayes factors (differences in log model evidence) at various values of SNR, where each point in each curve was averaged over 50 simulated data sets. For these DCM simulations, the averaging was implemented using the median operator (rather than the mean) as the results were more variable than for the GLM case. The curves in Fig. 7 show that only the Free Energy criterion is able to correctly identify the full model.

**Fig. 8:**
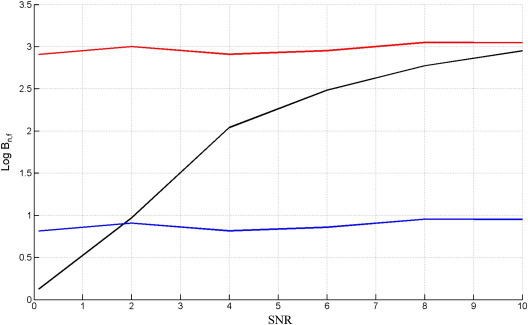
Log Bayes factor of nested versus full model, Log *B*_*n*, *f*_, versus the signal to noise ratio, SNR, when the true model is the nested DCM for *F*_*L*_ (black), AIC (blue) and BIC (red).

The above procedure was then repeated but this time generating data from the nested model. Again, each point in each curve is the median value over 50 simulated data sets. The results are shown in Fig. 8 (note the broader range of SNRs plotted).

The results on data from the nested model are very similar to those for the GLM case (compare Figs. 3 and 8). The results for data from the full model, however, are not (compare Figs. 2 and 7), as AIC and BIC are unable to correctly identify the full model even at high SNR. In order to find out why this is the case we examined DCMs fitted to data at SNR = 2, and examined the relative contributions to the model evidence, as described in Decompositions section.

For this high SNR scenario we found, slightly to our surprise, that the full DCMs were only slightly more accurate than the nested DCMs. Unsurprisingly, this increase in accuracy was realised in region A, which receives modulatory input in the full but not in the nested model (see Fig. 6). However, the main quantity driving the difference in Free Energy between full and nested DCMs was not the accuracy but rather the complexity.

It turns out that the nested DCMs are able to produce a reasonable data fit by using a very large value for the intrinsic connection, *a*_*AF*_ (from region F to A). This connection value (typically 1.5) was about 5 times bigger than the value for a full DCM (typically 0.3). This makes sense because, in the nested model, the connection from P to F is modulated by intelligibility, and by facilitating the intrinsic connection from F to A this ‘modulatory signal’ is passed on to region A. Since this modulation is of an additive nature, this therefore crudely mimics a direct modulation of the P to A connection. However, such a strong intrinsic connection from F to A is a-priori unlikely (the prior is a zero-mean Gaussian, with standard deviation *σ*_*cross*_ = 0.5). The nested models are therefore heavily penalised for having such unlikely parameter values (being three standard deviations away from their prior means). Only the Free Energy criterion is sensitive to such subtleties because AIC and BIC pay the same penalty for each parameter, regardless of magnitude.

As mentioned above, the empirical SNR for this data is SNR = 0.2 which is very low. Fitting the full and nested DCMs to this data yielded a Free Energy difference of only 0.11 (in favour of the full DCM). This difference is negligible, and points to the difficulty of model inference for very similar models and at low SNR (as exemplified by Figs. 7 and 8). In this regime it may be a better idea to make inferences over families of models (Penny et al., 2010) and to look for consistent differences over a group of subjects (Stephan et al., 2009).

## Discussion

We have described a simulation study which compared the relative merits of AIC, BIC and Free Energy model selection criteria. Differences in performance were examined in the context of GLMs and DCMs and we found that the Free Energy has the best model selection ability and recommended it be used for comparison of DCMs. Similar conclusions have been reached in earlier work comparing Free Energy with BIC in the context of non-Gaussian autoregressive modelling (Roberts and Penny, 2002) and Hidden Markov Modelling (Valente and Wellekens, 2004).

The GLM simulation results showed that, at low SNR, AIC and BIC incorrectly selected nested models when data were generated by full models. At higher SNR, however, this bias disappeared and AIC/BIC showed increased sensitivity. We also investigated a corrected AIC criterion but this showed no benefit over the standard AIC measure.

The DCM simulation results showed that only the Free Energy was able to correctly detect that data had been generated from the full model. By decomposing the Free Energy difference into contributions from different regions and parameters, we found that this ability was mainly due to penalising the nested model for having a very large, and a-priori unlikely, intrinsic connection from brain region F to A. Because AIC and BIC use the same complexity penalty for every parameter, and one that is not matched to prior expectations, they lack the sensitivity that is required, in this case, to infer that data was drawn from the full model.

We emphasise that this will not always be the case, and AIC/BIC can in general be sensitive to ‘full model’ effects in DCMs. This is demonstrated, for example, in our previous work (Penny et al., 2004). However, if prior information about parameter values is available then it should be used, and can be used to good effect in the Free Energy criterion.

It may also be argued that in the application in this paper AIC and BIC are implicitly using prior information in that the accuracy term is computed at the maximum posterior value. Being a posterior estimate this is naturally constrained by the prior. To avoid this one would have to implement a separate Maximum Likelihood optimisation. Given this fact, it therefore seems consistent to also use prior information when approximating the evidence.

According to conventions in Bayesian statistics (Kass and Raftery, 1995), and as stated above, models can be considered clearly distinguishable once the log Bayes factor exceeds three. The simulation results for both GLMs and DCMs show smaller Bayes factors when the true model is nested rather than full. This is particularly pronounced for the (challengingly similar) DCMs examined in this paper for which the Free Energy only achieves a Log Bayes Factor of three at an SNR of 10. In such a case, modellers and imaging neuroscientists should appeal to a second Occam principle (Mackay, 2003), not the numerical one embedded in the equation for the Free Energy, but a conceptual one that when two models cannot be clearly distinguished one should prefer the simpler one.

In previous work (Penny et al., 2004) we have advocated the combined use of AIC and BIC criteria for the comparison of DCMs. This was motivated by a concern about how Free Energy model inference depends on the chosen values of the prior means and variances (see earlier section on priors). Specifically, the values *σ*_*self*_, *σ*_*cross*_ and *σ*_*s*_ implicitly set the penalty paid for intrinsic, modulatory and input parameters (as governed by Eq. (21) via the overall prior covariance matrix *C*_*θ*_.).

This therefore motivates the future application of an Empirical Bayes (Carlin and Louis, 2000) approach which would estimate these variance parameters from data. This would effectively perform a search in the continuous space of prior variances instead of the discrete space (e.g., nested versus full) examined in this paper. Such an approach can be implemented within the new framework of post-hoc model selection (Friston and Penny, 2011).

## Appendix A Hemodynamics

In DCM, neuronal activity gives rise to fMRI activity by a dynamic process described by an extended Balloon model (Buxton et al., 2004) and BOLD signal model (Stephan et al., 2007) for each region. This specifies how changes in neuronal activity give rise to changes in blood oxygenation that are measured with fMRI.

The hemodynamic model involves a set of hemodynamic state variables, state equations and hemodynamic parameters. For the *i*th region, neuronal activity *z(i)* causes an increase in vasodilatory signal *s*_*i*_ that is subject to autoregulatory feedback. Inflow *f*_*i*_ responds in proportion to this signal with concomitant changes in blood volume *v*_*i*_ and deoxyhemoglobin content *q*_*i*_.

(35) $$\begin{array}{c} {\dot{s}}_{i}=z(i)-\kappa_{i}s_{i}-\gamma (f_{i}-1)\\ {\dot{f}}_{i}=s_{i}\\ \tau_{i}{\dot{v}}_{i}=f_{i}-v_{i}^{1/\alpha }\\ \tau_{i}{\dot{q}}_{i}=f_{i}\frac{E(f_{i},\rho )}{\rho }-v_{i}^{1/\alpha }\frac{q_{i}}{v_{i}} \end{array}$$

Outflow is related to volume *f*_*out*_ = *v*^1/*α*^ through Grubb's exponent *α* (Friston et al., 2003). The oxygen extraction is a function of flow

(36) $$E(f,\rho )=1-{\left(1-\rho \right)}^{1/f}$$

where *ρ* is resting oxygen extraction fraction. The free parameters of the model are the rate of signal decay in each region, *κ*_*i*_, and the transit time in each region, *τ*_*i*_. The other parameters are fixed to *γ* = *α* = *ρ* = 0.32.

### A.1 BOLD signal model

The Blood Oxygenation Level Dependent (BOLD) signal is then taken to be a static nonlinear function of volume and deoxyhemoglobin that comprises a volume-weighted sum of extra- and intra-vascular signals. This is based on a simplified approach from Stephan et al. (Stephan et al., 2007) (Eq. 12) that improves upon the earlier model (Friston et al., 2003)

(37) $$\begin{array}{c} y_{i}=V_{0}\left[k_{1}(1-q_{i})+k_{2}(1-\frac{q_{i}}{v_{i}})+k_{3}(1-v_{i})\right]\\ k_{1}=4.3\theta_{0}\rho TE\\ k_{2}=\varepsilon r_{0}\rho TE\\ k_{3}=1-\varepsilon \end{array}$$

where *V*_0_ is resting blood volume fraction, *θ*_0_ is the frequency offset at the outer surface of the magnetised vessel for fully deoxygenated blood at 1.5T, TE is the echo time and *r*_0_ is the slope of the relation between the intravascular relaxation rate and oxygen saturation (Stephan et al., 2007). In this paper we use the standard parameter values *V*_0_ = 4, *r*_0_ = 25, *θ*_0_ = 40.3 and for our fMRI imaging sequence we have *TE* = 0.04.

The only free parameter of the BOLD signal model is *ε*, the ratio of intra- to extra-vascular signal. Together the above equations describe a nonlinear hemodynamic process and BOLD signal model that convert neuronal activity in the *i*th region, *z*_*i*_, to the fMRI signal, *y*_*i*_.

### A.2 Priors

The unknown parameters are {*κ*_*i*_, *τ*_*i*_, *ε*}. These are represented as

(38) $$\begin{array}{c} \kappa_{i}=0.64exp(\theta_{\kappa_{i}})\\ \tau_{i}=2exp(\theta_{\tau_{i}})\\ \varepsilon =exp(\theta_{\varepsilon }) \end{array}$$

and we have Gaussian priors

(39) $$\begin{array}{c} p(\theta_{\kappa_{i}})==\text{N}(\theta_{\kappa_{i}};0,0.135)\\ p(\theta_{\tau_{i}})==\text{N}(\theta_{\tau_{i}};0,0.135)\\ p(\varepsilon )==\text{N}(\varepsilon ;0,0.135) \end{array}$$

where *h* = {*θ*_*κ*_*i*__, *θ*_*τ*_*i*__, *ε*} are the hemodynamic parameters to be estimated.

## Appendix B Laplace approximation

In what follows we have simplified notation by dropping the dependence on model *m*. The negative variational free energy (henceforth ‘Free Energy’) is defined as

(40) $$F=\int \int q(\theta |y)q(\lambda |y)log\left[\frac{p(y,\theta ,\lambda )}{q(\theta |y)q(\lambda |y)}\right]\;d\theta d\lambda$$

where

(41) p(y,θ,λ)=p(y|θ,λ)p(θ)p(λ)

We can rewrite this as

(42) F=I+H(θ)+H(λ)

where

(43) I=∫∫q(θ|y)q(λ|y)U(θ,λ)dθdλ

and *H(x)* is the (differential) entropy of *x* and

(44) U(θ,λ)=logp(y,θ,λ)

For a Gaussian density *p*(*x*) = *N*(*x* ; *m*, *S*) the entropy is

(45) $$H(x)=\frac{1}{2}\left(klog2\pi e+log|S|\right)$$

where *k* = *dim*(*S*). Hence

(46) $$\begin{array}{l} F=I+\frac{1}{2}\left(plog2\pi e+log|S_{\theta }|\right)\\ +\frac{1}{2}\left(hlog2\pi e+log|S_{\lambda }|\right) \end{array}$$

where *p* is the number of parameters and *h* is the number of hyperparameters. The Variational Laplace approximation to the Free Energy is then given by

(47) $$\begin{array}{l} F_{L}=I_{L}+\frac{1}{2}\left(plog2\pi e+log|S_{\theta }|\right)\\ +\frac{1}{2}\left(hlog2\pi e+log|S_{\lambda }|\right) \end{array}$$

where the integral *I* has been replaced by

(48) $$I_{L}=\int \int q(\theta |y)q(\lambda |y)U_{L}(\theta ,\lambda )d\theta d\lambda$$

and the function *U*_*L*_(*θ*, *λ*) is given by a second order Taylor series expansion around the approximate (variational) posterior means

(49) $$\begin{array}{l} U_{L}(\theta ,\lambda )=U(m_{\theta },m_{\lambda })+\frac{1}{2}\;{\left(\theta -m_{\theta }\right)}^{T}H_{\theta }(\theta -m_{\theta })\\ +\frac{1}{2}\;{\left(\lambda -m_{\lambda }\right)}^{T}H_{\lambda }(\lambda -m_{\lambda }) \end{array}$$

where the curvatures

(50) $$\begin{array}{l} H_{\theta }(i,j)=\frac{d^{2}U(\theta ,\lambda )}{d\theta_{i}d\theta_{j}}\\ H_{\lambda }(i,j)=\frac{d^{2}U(\theta ,\lambda )}{d\lambda_{i}d\lambda_{j}} \end{array}$$

are evaluated at the approximate (variational) posterior means *λ* = *m*_*λ*_ and *θ* = *m*_*θ*_. Note that the first order (gradient) term in Eq. (49) is zero because we are at a maximum. This gives

(51) $$I_{L}=U(m_{\theta },m_{\lambda })+\frac{1}{2}Tr(S_{\theta }H_{\theta })+\frac{1}{2}Tr(S_{\lambda }H_{\lambda })$$

During VL optimisation (Friston et al., 2007a) the posterior covariances are set to the negative inverse curvatures

(52) $$\begin{array}{l} S_{\theta }=-H_{\theta }^{-1}\\ S_{\lambda }=-H_{\lambda }^{-1} \end{array}$$

Hence

(53) $$I_{L}=U(m_{\theta },m_{\lambda })-\frac{p}{2}-\frac{h}{2}$$

Substituting this into Eq. (47) gives

(54) $$\begin{array}{l} F_{L}=U(m_{\theta },m_{\lambda })+\frac{p}{2}log2\pi +\frac{1}{2}log|S_{\theta }|\\ +\frac{p}{2}log2\pi +\frac{1}{2}log|S_{\lambda }| \end{array}$$

This corresponds to equation 8 in (Friston et al., 2007a). We note that

(55) $$\begin{array}{l} U(m_{\theta },m_{\lambda })=logp(y|m_{\theta },m_{\lambda })p(m_{\theta })p(m_{\lambda })\\ =-\frac{1}{2}e_{y}^{T}C_{y}^{-1}e_{y}-\frac{1}{2}log|C_{y}|-\frac{N}{2}log2\pi \\ -\frac{1}{2}e_{\theta }^{T}C_{\theta }^{-1}e_{\theta }-\frac{1}{2}log|C_{\theta }|-\frac{p}{2}log2\pi \\ -\frac{1}{2}e_{\lambda }^{T}C_{\lambda }^{-1}e_{\lambda }-\frac{1}{2}log|C_{\lambda }|-\frac{h}{2}log2\pi \end{array}$$

where the error terms are

(56) $$\begin{array}{l} e_{y}=y-g(m_{\theta })\\ e_{\theta }=m_{\theta }-\mu_{\theta }\\ e_{\lambda }=m_{\lambda }-\mu_{\lambda } \end{array}$$

Finally, we have

(57) $$\begin{array}{l} F_{L}=-\frac{1}{2}e_{y}^{T}C_{y}^{-1}e_{y}-\frac{1}{2}log|C_{y}|-\frac{N}{2}log2\pi \\ -\frac{1}{2}e_{\theta }^{T}C_{\theta }^{-1}e_{\theta }-\frac{1}{2}log|C_{\theta }|+\frac{1}{2}log|S_{\theta }|\\ -\frac{1}{2}e_{\lambda }^{T}C_{\lambda }^{-1}e_{\lambda }-\frac{1}{2}log|C_{\lambda }|+\frac{1}{2}log|S_{\lambda }| \end{array}$$

This corresponds to equation 21 in (Friston et al., 2007a).

The quantity *U*_*L*_(*θ*, *λ*) is equal to *U*(*θ*, *λ*) if the latter is a quadratic function. This is the case for linear Gaussian models. For all other models, where the quadratic relationship does not hold exactly, *U*_*L*_ can be bigger or smaller than *U*. For this reason *F*_*L*_ can be bigger or smaller than *F*, so *F*_*L*_ is not a lower bound on the log model evidence (Wipf and Nagarajan, 2009).

## Appendix C Proportion of variance explained

The proportion of variance explained by a model can be written as

(58) $$R^{2}=\frac{\sigma_{y}^{2}}{\sigma_{y}^{2}+\sigma_{e}^{2}}$$

where *σ*_*y*_^2^ is the variance of the signal and *σ*_*e*_^2^ is the variance of the noise. The left hand side is written with the symbol *R*^2^ because *R* is also equal to the correlation coefficient between the model predictions and data (Kleinbaum et al., 1988). We can divide the numerator and denominator by *σ*_*y*_^2^ to give

(59) $$R^{2}=\frac{1}{1+\frac{\sigma_{e}^{2}}{\sigma_{y}^{2}}}$$

Plugging in our definition for SNR gives

(60) $$R^{2}=\frac{1}{1+{\left(\frac{1}{SNR}\right)}^{2}}$$

Thus, SNRs of 0.2, 1.3 and 2 correspond to *R*^2^'s of 0.04, 0.63, and 0.80.

## References

[bb0005] Akaike H. (1973). Information measures and model selection. Bull. Int. Stat. Inst..

[bb0010] Attias H. (1999). Inferring parameters and structure of latent variable models by variational Bayes. Proceedings of the Fifteenth Conference on Uncertainty in Artificial Intelligence.

[bb0015] Beal M., Ghahramani Z., Bernardo J., Bayarri M., Berger J., Dawid A. (2003). The variational Bayesian EM algorithms for incomplete data: with application to scoring graphical model structures.

[bb0020] Bernardo J.M., Smith A.F.M. (2000). Bayesian Theory.

[bb0025] Bishop C.M. (2006). Pattern Recognition and Machine Learning.

[bb0030] Brockwell P., Davis R. (2009). Time Series: Theory and Methods.

[bb0035] Burnham K., Anderson D. (2002). Model selection and multimodel inference: a practical information theoretic approach.

[bb0040] Burnham K., Anderson D. (2004). Multimodel inference: understanding AIC and BIC in model selection. Sociol. Methods Res..

[bb0045] Buxton R., Uludag K., Dubowitz D., Liu T. (2004). Modelling the hemodynamic response to brain activation. NeuroImage.

[bb0050] Carlin B.P., Louis T.A. (2000). Bayes and Empirical Bayes Methods for Data Analysis.

[bb0055] Daunizeau J., Kiebel S.J., Friston K.J. (2009). Dynamic causal modelling of distributed electromagnetic responses. NeuroImage.

[bb0060] Friston K., Mattout J., Trujillo-Barreto N., Ashburner J., Penny W. (2007). Variational free energy and the Laplace approximation. NeuroImage.

[bb0065] Friston K., Penny W. (2011). Post hoc Bayesian model selection. NeuroImage.

[bb0070] Friston K.J. (2002). Bayesian estimation of dynamical systems: an application to fMRI. NeuroImage.

[bb0075] Friston K.J., Ashburner J., Kiebel S.J., Nichols T.E., Penny W.D. (2007). Statistical Parametric Mapping: The Analysis of Functional Brain Images.

[bb0080] Friston K.J., Harrison L., Daunizeau J., Kiebel S.J., Phillips C., Trujillo-Bareto N., Henson R.N.A., Flandin G., Mattout J. (2008). Multiple sparse priors for the M/EEG inverse problem. NeuroImage.

[bb0085] Friston K.J., Harrison L., Penny W.D. (2003). Dynamic causal modelling. NeuroImage.

[bb0090] Gelman A., Carlin J.B., Stern H.S., Rubin D.B. (1995). Bayesian Data Analysis.

[bb0095] Henson R.N.A., Shallice T., Gorno-Tempini M.L., Dolan R.J. (2002). Face repetition effects in implicit and explicit memory tests as measured by fMRI. Cereb. Cortex.

[bb0100] Hoeting J.A., Madigan D., Raftery A.E., Volinsky C.T. (1999). Bayesian model averaging: a tutorial. Stat. Sci..

[bb0105] Hurvich C., Tsai C. (1989). Regression and time series model selection in small samples. Biometrika.

[bb0110] Kass R.E., Raftery A.E. (1995). Bayes factors. J. Am. Stat. Assoc..

[bb0115] Kleinbaum D.G., Kupper L.L., Muller K.E. (1988). Applied Regression Analysis and Other Multivariable Methods.

[bb0120] Leff A., Schofield T., Stephan K., Crinion J., Friston K., Price C. (2008). The cortical dynamics of intelligible speech. J. Neurosci..

[bb0125] Mackay D.J.C. (2003). Information Theory, Inference and Learning Algorithms.

[bb0130] M. Beal. Variational Algorithms for Approximate Bayesian Inference. PhD thesis, Gatsby Computational Neuroscience Unit, University College London, 2003.

[bb0135] Penny W.D., Kiebel S.J., Friston K.J. (2003). Variational Bayesian Inference for fMRI time series. NeuroImage.

[bb0140] Penny W.D., Roberts S.J. (2002). Bayesian multivariate autoregresive models with structured priors. IEE Proc. Vis., Image Signal Process..

[bb0145] Penny W.D., Stephan K.E., Daunizeau J., Rosa M.J., Friston K.J., Schofield T.M., Leff A.P. (2010). Comparing families of dynamic causal models. PLoS Comput. Biol..

[bb0150] Penny W.D., Stephan K.E., Mechelli A., Friston K.J. (2004). Comparing dynamic causal models. NeuroImage.

[bb0155] Raftery A.E., Marsden P.V. (1995). Bayesian model selection in social research. Sociological Methodology.

[bb0160] Roberts S.J., Penny W.D. (2002). Variational Bayes for generalised autoregressive models. IEEE Trans. Signal Process..

[bb0165] Schwarz G. (1978). Estimating the dimension of a model. Ann. Stat..

[bb0170] Stephan K., Penny W., Daunizeau J., Moran R.J., Friston K.J. (2009). Bayesian model selection for group studies. NeuroImage.

[bb0175] Stephan K., Weiskopf N., Drysdale P., Robinson P., Friston K. (2007). Comparing hemodynamic models with DCM. NeuroImage.

[bb0180] Stephan K.E., Penny W.D., Moran R.J., den Ouden H.E.M., Daunizeau J., Friston K.J. (2010). Ten simple rules for dynamic causal modeling. NeuroImage.

[bb0185] Trujillo-Barreto N., Aubert-Vazquez E., Valdes-Sosa P.A. (2004). Bayesian model averaging in EEG/MEG imaging. NeuroImage.

[bb0190] Valente F., Wellekens C. (2004). Scoring unknown speaker clustering: VB vs BIC. ICSLP 2004, 8th Biennial conference of International Conference on Spoken Language Processing.

[bb0195] Wipf D., Nagarajan S. (2009). A unified Bayesian framework for MEG/EEG source imaging. NeuroImage.

